# Impact of Manual Therapy on Plantar Pressures in Patients with Fibromyalgia: A Single-Arm, Non-Randomized Pilot Clinical Trial

**DOI:** 10.3390/healthcare13070764

**Published:** 2025-03-29

**Authors:** Francisco J. Falaguera-Vera, Javier Torralba-Estellés, Juan Vicente-Mampel, Javier Ferrer-Torregrosa, Elisa Oltra, María Garcia-Escudero

**Affiliations:** 1 Department of Physiotherapy, School of Medicine and Health Science, Catholic University of Valencia, Torrent, 46001 Valencia, Spain; fj.falaguera@ucv.es (F.J.F.-V.); juan.vicente@ucv.es (J.V.-M.); maria.escudero@ucv.es (M.G.-E.); 2 Department of Podiatry, School of Medicine and Health Science, Catholic University of Valencia, Torrent, 46001 Valencia, Spain; javier.torralba@ucv.es; 3 Department of Pathology, School of Medicine and Health Sciences, Universidad Católica de Valencia San Vicente Mártir, 46001 Valencia, Spain; elisa.oltra@ucv.es

**Keywords:** fibromyalgia, plantar pressures, physical therapy, baropodometry, quality of life, gait, body mass index, manual therapy, chronic pain, multidisciplinary intervention

## Abstract

Background: Fibromyalgia (FM) is a chronic disorder causing widespread musculoskeletal pain, often leading to physical deconditioning that affects posture and gait. This study evaluates the effects of a manual therapy protocol targeting dorsal muscles in the lower back on plantar pressure modifications, considering body mass index (BMI) influence. Methods: A single-arm, non-randomized clinical trial included 24 women diagnosed with FM for at least three years. They underwent an eight-session manual therapy protocol over four weeks, applying moderate pressure to dorsal muscles in the lower back. Baropodometric analyses were conducted pre- and post-intervention under dynamic conditions. Statistical analyses used paired *t*-tests and effect size calculations to assess intervention effects and BMI impact. Results: Significant improvements in plantar pressure distribution were observed in both the left foot (*p* = 0.01, d = −0.54) and the right foot (*p* = 0.008, d = −0.59). However, strength and peak pressure metrics showed no significant changes. Patients with normal BMI exhibited greater improvements than those in the overweight category. Conclusions: Preliminary findings suggest that manual therapy positively influenced plantar pressure distribution in FM patients, particularly in those with normal BMI. Further research is needed to explore long-term effects and broader clinical applications.

## 1. Introduction

Fibromyalgia (FM) is a chronic and complex disorder that affects approximately 3–10% of the world’s population [1], with a significantly higher prevalence in women than in men [2]. FM occurs more frequently in women between 20 and 55 years of age and shows an increase with advancing age, affecting approximately 7.4% of women between 70 and 79 years of age [3]. It is characterized by widespread musculoskeletal pain that persists for at least three months, marked by various indicators, including exhaustion [4], impaired mental function, and disrupted sleep patterns [5], in addition to other debilitating symptoms such as gastrointestinal problems, anxiety, and depression [4]. The exact cause of FM remains unknown, but multiple factors contribute to its development [6]. These include genetic predisposition [7], imbalances in neurotransmitters such as serotonin and dopamine, changes in cytokine profiles (both pro- and anti-inflammatory), alterations in growth hormone levels, and dysfunction in pain processing within the central nervous system [8]. This dysfunction, known as central sensitization, amplifies pain signals [2], leading to heightened sensitivity to stimuli that are typically not painful [9]. Clinically, FM symptoms are closely linked to central sensitization [10,11], with patients often experiencing increased pain sensitivity (hyperalgesia) and widespread pain from non-painful stimuli (allodynia) [4].

Diagnosing FM remains challenging because of the absence of specific laboratory or imaging tests. Traditionally, diagnosis relies on clinical criteria established by the American College of Rheumatology (ACR) in 1990 [12], with subsequent updates, including the 2016 revisions to the 2010/2011 criteria [1,13,14], which improved diagnostic accuracy by evaluating widespread pain and associated symptoms. The treatment of FM primarily aims to alleviate symptoms and improve the quality of life of patients through a multidisciplinary approach. physical therapy [15,16,17], moderate exercise, and cognitive-behavioral therapy play crucial roles alongside pharmacological options such as analgesics and antidepressants [18,19,20,21,22,23]. Given the significant impact of chronic fatigue and pain on daily activities [24,25], work performance, and social interactions, early diagnosis and comprehensive treatment are essential to enhance clinical outcomes and reduce the associated economic burden [18,19,20]. Effective pain management strategies must focus on the chronic nature of FM, prioritizing approaches that offer sustainable relief and improve functionality. Central sensitization is a key etiopathological factor in FM, contributing to widespread pain and physical dysfunction [26,27,28]. This condition is associated with motor control alterations, physical deconditioning, and muscle weakness, which collectively impair postures, balance, and gait [29,30]. Such impairments can lead to abnormal body weight distribution and altered loading patterns during static and dynamic stances [31,32]. In patients with FM, recent studies have confirmed increased plantar pressure, particularly under the metatarsal heads and heel, resulting in an asymmetric load distribution between both feet [33,34,35]. This uneven pressure distribution often results in increased forefoot pressure peaks while walking or excessive heel loading when standing, thereby reducing the foot’s natural cushioning capacity [35,36]. This condition significantly contributes to pain, a higher risk of falls, and substantial disability [36,37]. Additionally, pain-related gait adaptations, such as slower walking speeds, shorter stride lengths, and prolonged stance phases, exacerbate these pressure imbalances, increasing the mechanical stress on specific foot regions [38]. These alterations may also contribute to greater foot fatigue, discomfort, and a higher risk of overuse injuries such as plantar fasciitis or stress fractures [39,40].

Although FM has been associated with postural and gait disturbances, its specific impact on foot mechanics and plantar pressure has not been thoroughly explored. Understanding these effects is essential for identifying potential functional impairments and developing targeted interventions for individuals with FM.

This study aimed to evaluate the impact of a manual therapy protocol targeting the posterior lumbar musculature on changes in plantar pressure in patients with FM, considering the influence of body mass index (BMI). The researchers hypothesized that this manual therapy approach would not only improve peak plantar pressures but also enhance the distribution of the plantar surface area, promoting a more balanced redistribution of foot pressures. Additionally, this study will explore whether BMI serves as a determining factor in the extent of improvement or potential deterioration in peak plantar pressure and plantar surface outcomes for both feet.

## 2. Materials and Methods

### 2.1. Study Design

This work was designed as a pilot, interventional, non-randomized, single-arm clinical trial, registered in ClinicalTrials.gov, and was approved with the objective of evaluating the effects of a manual therapy (MT) protocol in patients with FM. The study had the approval of the Ethics Committee UCV/2018-2019/076, complying with the ethical principles established in the Declaration of Helsinki [41]. The study design and progression of the participants were conducted in accordance with the CONSORT guidelines [42].

### 2.2. Participants

The sample included 35 female patients diagnosed with FM who voluntarily participated in the study after providing written informed consent [41,43]. Measurements were performed before and after the implementation of manual therapy (MT) to analyze changes in plantar pressure and foot surface area, considering factors such as BMI.

Participants met the ACR criteria, as indicated by their local medical center certificate [12,13]. They were also asked about a possible comorbid diagnosis of myalgic encephalomyelitis/chronic fatigue syndrome (ME/CFS) according to the Canadian and/or international criteria for ME/CFS [7,44]. Participants were recruited between January and February 2019 through invitations from several associations of patients with FM in the Valencian Community. Informative talks were held in various FM associations in the Valencian Community, aimed mainly at patients diagnosed with FM and their families.

During these sessions, the objectives of the study, methodology, and expected results were presented, and an information sheet with the requirements to participate in the research was provided. The approach was to ensure diverse and representative participation of the community, facilitating access to information and resolving doubts of the attendees. Appointments were assigned individually to each participant to carry out the entire process of the study, which was conducted in the clinical trial room of the Catholic University of Valencia between March and April 2019. To ensure the stability and chronicity of FM among the participants, the study required a confirmed diagnosis of FM for at least 3 years. This criterion was intended to minimize the variability associated with newly diagnosed patients who may still be in the early stages of adaptation or treatment of their disease. By selecting individuals with more advanced and stable diseases, the study achieved a more homogeneous sample in terms of symptom progression and treatment history, which increased the reliability of the results. The inclusion criteria specified that participants had to (i) be older than 18 years, (ii) have a previous diagnosis of FM confirmed by a specialist, (iii) not have participated in recent drug trials, and (iv) abstain from drug use 12 h before the intervention. The exclusion criteria were as follows: (i) undergoing physiotherapeutic treatments incompatible with the study protocol, (ii) receiving hormonal treatments, and (iii) having a history of serious diseases such as cancer.

### 2.3. Intervention

#### 2.3.1. Manual Therapy Protocol

The physiotherapy protocol was designed based on existing evidence of the efficacy of MT in FM [15,45,46] and data on the effects of applied pressure in MT [47,48]. This included a total of eight sessions distributed over four weeks, with two weekly sessions of 25 min each. Sessions were scheduled on Mondays and Thursdays or Tuesdays and Fridays, with patients assigned to groups to ensure uniformity in days and times, as well as in environmental and basal conditions. The treatment was performed with the patients in the prone position, exposing the back and upper gluteal region. Massage therapy techniques were applied with moderate pressure, focusing on the back musculature (lumbar, dorsal and cervical regions, in their posterior and lateral portions) and the gluteal region (gluteus maximus, medius, and minimums), with emphasis on the pyramidal area.

#### 2.3.2. Treatment Sequence

Massage began on the dorsal and lumbar paravertebral musculature, followed by the posterior and lateral regions of the cervical spine, including the sternocleidomastoid (“Low cervical” pressure point) and the suboccipital insertion (“occiput” pressure point). Subsequently, the upper fibers of the trapezius (pressure point “trapezius”) were treated, continuing with the scapular region, where the supraspinatus (pressure point “supraspinatus”), infraspinatus, teres major, and teres minor muscles were manipulated. Finally, the latissimus dorsi, quadratus lumborum, and upper and outer gluteal regions (“gluteal” pressure point) were addressed. Each session consisted of two full rounds of this protocol, with 30–40 passes on each muscle. This controlled repetition helps achieve an optimal balance between muscle relaxation and activation of local blood flow, which is essential for reducing tension and improving tissue oxygenation. Moreover, the multiple passes contribute to decreasing the hypersensitivity of tender points, a characteristic symptom of FM, without overloading the tissues [46].

#### 2.3.3. Technique Applied

The physiotherapy treatment protocol consisted of eight sessions over four weeks, with two 25-min sessions per week. To maintain standardization, sessions were scheduled consistently (Monday-Thursday or Tuesday-Friday) under similar environmental conditions. The therapy consisted of moderate pressure massage techniques (approximately 4.5 N) applied to the muscles of the back (lumbar, dorsal, and cervical regions on both posterior and lateral sides) and the gluteal area. The target muscles were the trapezius (upper, middle and lower fibers), sternocleidomastoid, supraspinatus, infraspinatus, rhomboid, teres major and minor, latissimus dorsi, quadratus lumborum, paravertebral muscles, thoracolumbar fascia, piriformis, and all gluteal muscles (maximus, medius and minimus). Specific areas are excluded from the treatment, such as the second rib, the epicondylar musculature of the elbow, the greater trochanter (although this region could indirectly benefit from treatment of the gluteal musculature) and the inner aspect of the knee.

The massage therapy sessions included massage maneuvers based on basic movements following the direction of the muscle fibers. These maneuvers were performed with broad, slow movements using moderate pressure (4.5 N) to avoid the occurrence of extracellular oedema and muscle damage induced by using higher massage pressure [48].

To ensure accurate application of 4.5 N pressure, the physiotherapist received specialized training in the use of an FDIX Force Gage algometer, with a capacity of up to 200 lbf/100 kgf/1000 N and an accuracy of ±0.2% for a single force cell module and ±0.3% for multiple modules. The device was fitted with a 1 cm^2^ rubber disc for applying pressure. device (FlexiForce, Tekscan, Canada). This device allowed accurate measurement and quantification of the applied load. The training process lasted five consecutive days, during which the physiotherapist practiced the protocol until three consecutive measurements were consistently within 10% of the target pressure. In addition, before each therapy session, a test was performed to verify the consistency of the applied techniques.

### 2.4. Measurements

#### 2.4.1. Baropodometric Analysis

The study assessed plantar pressure utilizing the Podoprint pressure platform (Grupo Namrol, Barcelona, Spain), a portable system measuring 570 × 570 mm, with a thickness of 9 mm and a weight of 3.8 kg. This platform features 1600 high-sensitivity sensors, capable of capturing up to 200 images per second and transmitting data to a connected computer for analysis.

**Figure 1:**
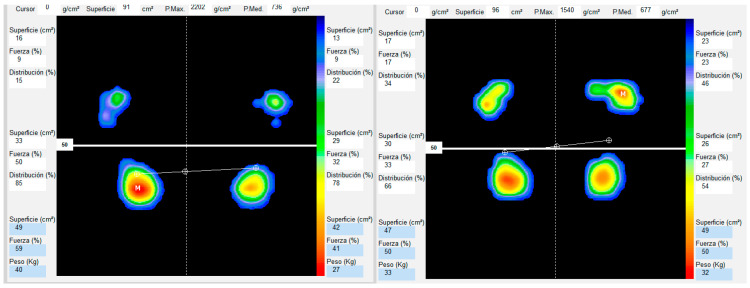
The image shows the Posturological analysis of a 50-year-old overweight woman diagnosed with FM. The colors represent different levels of plantar pressure on the support surface: red indicates areas of higher pressure, and blue indicates areas of lower pressure. The quantitative data used in this study can be observed around the image.

Prior to and following the MT treatment sequence, participants underwent a postural examination to obtain a quantitative mapping of plantar pressures and the support surface [49,50]. To minimize measurement bias, all participants performed the test barefoot, and the equipment was calibrated before each session in accordance with the manufacturer’s guidelines. During the assessment, each participant stepped onto the Podoprint ® platform (Medicapteurs, Blama, France) and assumed a comfortable position, maintaining their individual Fick angle. They were instructed to remain stationary for 10 s, allowing for a stable measurement of pressure distribution. The software provided a detailed visualization of the plantar footprint, utilizing a color-coded scale where warm colors (red, orange, yellow) indicate high-pressure zones, while cool colors (blue, green) represent areas with lower pressure. Additional parameters, such as load distribution and support surface, were also analyzed. To ensure reliable and consistent results, three assessments were conducted under standardized conditions, and the mean of these measurements was used for the final analysis [51,52]. This protocol enabled an objective evaluation of pressure distribution and force percentage for each foot (See Figure 1).

#### 2.4.2. Variables

BMI was classified as follows: a value below 18.5 indicates underweight; between 18.5 and 24.9 corresponds to a normal or healthy weight; and between 25.0 and 29.9 is considered to be in the overweight range [53].

Plantar pressure Max (g/cm^2^): Measures the force exerted by the foot on the ground at each point of contact, expressed in grams per square centimeter.

Foot-bearing surface (cm^2^): Represents the total area of the foot in contact with the ground during the static position, measured in square centimeters.

Foot Force (%): The percentage of total body weight supported by each foot when the person is in a static standing position.

### 2.5. Sample Size Calculation

The sample size calculation, based on a moderate effect size (Cohen’s d = 0.6), a 5% significance level (α = 0.05), and an 80% statistical power (1 − β = 0.8), determined the need for 24 participants, which aligned with the sample size included in this pilot study.

### 2.6. Statistical Analysis

Analyses were performed by an external observer who was blinded to the experimental design. Data are presented as mean ± standard deviation (SD). The normality of the data was assessed using the Kolmogorov–Smirnov test, and the homogeneity of variances was tested using Levene’s test. A significance level of *p* > 0.05 was established. Statistical analyses were performed using SPSS version 24 (SPSS Inc., Chicago, IL, USA), and graphical representations were generated using JASP software (v0.16.4, Amsterdam, The Netherlands).

A comparative analysis of pre- and post-intervention measurements was performed to assess the changes in the selected variables. Paired *t*-tests (Wilcoxon when data did not meet normality) were used to analyze differences, and effect sizes (Cohen’s d) were calculated to interpret the effect size. Effect sizes were classified into the following categories: trivial (<0.20), small (0.20–0.59), moderate (0.60–1.19), large (1.20–1.99) and very large (>2.00) [54].

To quantify the changes resulting from the intervention, the “Delta” variable was calculated as the difference between each participant’s post-intervention and pre-intervention measurements (Delta = Post value − pre value). These Deltas were then compared across groups classified by BMI.

## 3. Results

### 3.1. Participation Flow and Sample Characteristics

**Figure 2:**
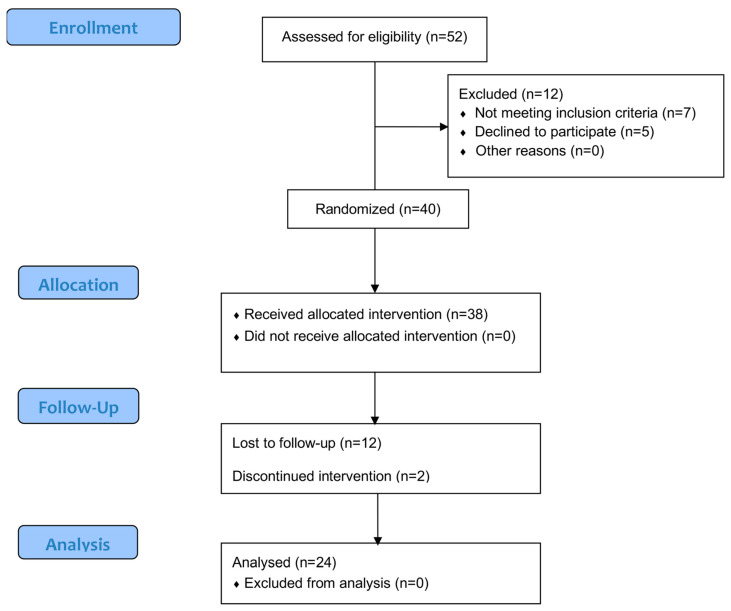
Flow Diagram.

Initially, 52 individuals were screened for eligibility, of whom 12 were excluded because they did not meet the criteria or refused to participate in the study. Forty participants were randomized. Of these, 38 received the assigned intervention, ensuring that no participant was left without treatment. During follow-up, 12 participants were lost, and 2 participants discontinued the intervention. Ultimately, 24 participants were included in the analysis phase, without any additional exclusions. The participants had a confirmed diagnosis of FM with a mean duration of 10.3 ± 7.5 years since the initial diagnosis (Figure 2).

Twenty-four women participated in the study. An independent samples *t*-test was used to examine the differences in the means of the anthropometric variables between the two BMI groups. The results showed no evidence of heterogeneity, and the analyses indicated the absence of significant differences between the groups (*p* > 0.05), except for BMI (Table 1).

### 3.2. Results of Baropodometric Analysis of the Total Sample Pre-Post

Student’s t analysis for paired samples allowed us to identify significant differences in some of the variables analyzed, while others showed no relevant changes between the pre- and post-intervention measurements. In the surface area variable, statistically significant differences were observed (t(23) = −3.01, *p* = 0.006), with a moderate effect size (d = −0.61, ET = 0.11). This indicates a significant reduction in the surface area after the intervention, which could reflect relevant changes in weight distribution or postural stability.

Left Foot Surface (LFS) also showed significant differences (t(23) = −2.65, *p* = 0.01), with a moderate effect size (d = −0.54, ET = 0.14). This suggests a decrease in the bearing surface of the left foot after the intervention, which could be related to modifications in the pressure distribution of the foot.

In contrast, Left Foot (LF) strength did not present statistically significant differences (t(23) = −0.20, *p* = 0.85), and the effect size was trivial (d = −0.04, ET = 0.19). This indicates that the intervention did not generate any appreciable changes in this specific variable.

In contrast, the Right Foot Surface (RFS) showed significant differences (t(23) = −2.91, *p* = 0.008), with a moderate effect size (d = −0.59, ET = 0.10). This reduction in the post-intervention right foot bearing surface may reflect similar adaptations to those observed in the left foot.

For Right Foot (RFT) strength, the results indicated no significant differences (t(23) = 0.20, *p* = 0.85), with a trivial effect size (d = 0.04, ET = 0.19). As with the left foot, strength did not seem to be affected in a relevant way by the intervention.

Finally, for Peak Pressure (P.Max), no statistically significant differences were detected (t(23) = 1.25, *p* = 0.22), and the effect size was small (d = 0.25, ET = 0.18). Overall, the results suggest that the intervention had a relevant impact on the variables related to the support surface, particularly in the measurements of both feet and the global surface, whereas the forces did not show significant changes. These findings may indicate specific postural adaptations rather than changes in the applied forces (See Table 2).

### 3.3. Results of Baropodometric Analysis Divided into BMI Groups

The analysis of the results indicated that the intervention generated significant effects on some variables, mainly in the group classified as “normal”, while no relevant changes were observed in the overweight group.

For the surface area variable, the normal group presented statistically significant differences (*p* = 0.004), with a moderate-large effect size (d = −0.92), indicating a significant reduction after the intervention. However, in the overweight group, the results showed no significant change (*p* = 0.57).

Similarly, in the Left Foot Surface (LFS), significant differences were identified in the normal group (*p* = 0.004), with a large effect size (d = −0.92), suggesting a relevant impact of the intervention on this variable.

In contrast, no differences were found in the overweight group (*p* = 0.94). Regarding the Right Foot Surface (RFS), the normal group also presented significant differences (*p* = 0.01), with a moderate effect size (d = −0.80), reflecting a significant reduction after the intervention. However, the overweight group did not show significant differences (*p* = 0.35).

In the variables related to strength (both Left and Right Foot) and Maximum Pressure (P.Max), no statistically significant differences were observed in any of the groups. Although some effect sizes were small to moderate, these changes were not sufficient to reach statistical significance.

The effects of the intervention appeared to be more marked in the group classified as “normal”, especially in the variables related to surface area, while the overweight group did not show significant changes in the metrics analyzed (See Table 3).

The results of the comparative analysis of the deltas of the variables between the groups according to BMI show that only some differences reached statistical significance, while others were not relevant (See Table 4).

In the Surface Delta, statistically significant differences were found (*p* = 0.05), with a moderate effect size (d = −0.85). This indicates a relevant difference in surface area changes between groups with different BMI categories.

In the Left Foot Surface Delta (LF), significant differences were also identified (*p* = 0.03), with a large effect size (d = −0.98). This suggests that the change in the left foot surface area differed between the groups, with one group showing greater change.

However, no significant differences were observed for Peak Pressure Delta (*p*. Max) and Right Foot Surface Delta (RF) (*p* = 0.49 and *p* = 0.21, respectively), with small effect sizes (d = −0.29 and d = −0.53). This indicates that the changes in these variables were similar between the groups.

For Mean Pressure Delta (P.Med) and Right Foot Force Delta (RF), although the results did not reach statistical significance (*p* = 0.09 and *p* = 0.14), the effect sizes (d = 0.74 and d = 0.64) were moderate, suggesting a trend toward differences in changes between groups.

In summary, the analyses indicated that the largest differences between the groups according to BMI were observed in the variables related to Surface Delta and Left Foot Surface Delta (LF), while the other variables did not reach statistical significance, although some showed notable trends (See Table 5).

## 4. Discussion

The results of this pilot study suggest that manual therapy directed at the posterior musculature may significantly improve the increase in foot contact surface with a more optimal distribution of plantar pressures in patients with FM. Specifically, changes were observed in plantar surface measurements, indicating enhanced stability and redistribution of body weight following the intervention. These findings support the initial hypothesis that treatment of the posterior musculature can positively influence plantar support patterns and posture, which is crucial in a population facing challenges related to chronic pain and biomechanical alterations. The results are consistent with previous studies highlighting the importance of physiotherapy and manual therapy in the treatment of FM symptoms [15,16,17]. Prior research has demonstrated that manual therapy treatment can reduce musculoskeletal pain and improve physical function in patients with FM [24,25], specifically in relation to plantar pressure distribution.

Foot deformities in individuals with FM are associated with an increased risk of falls [37,55] and significantly impact plantar pressures and postural stability. In this context, foot problems are considered risk factors that may limit patients’ mobility, as they contribute to altered static pressure distribution, potentially increasing discomfort and postural imbalances.

The complexity associated with FM presents challenges in establishing a singular effective treatment. Consequently, the therapeutic approach is multidisciplinary, encompassing both pharmacological and non-pharmacological interventions aimed at symptom alleviation [56]. Manual therapy has been identified as efficacious for treating FM. However, the study supporting this treatment approach exhibits limitations of studies included [57]. Additionally, combined exercise, aquatic exercise, and other active therapies have demonstrated improvements in pain intensity, disability, and physical function in the short term [58]. Furthermore, the application of a manual therapy protocol has been found to be effective in ameliorating pain and posture [59]. Overall, evidence substantiates the positive effect of physiotherapy treatments, including manual therapy, on the signs and symptoms of FM, such as pain, physical impairment, and reduced quality of life [25].

Research specifically examining the connection between plantar pressures and FM is scarce [33]. Although various biomechanical changes are observed in FM patients, a prevalent characteristic is the pronated foot position, which is frequently observed in individuals with FM. This postural anomaly could significantly impact overall body biomechanics, as incorrect foot alignment may lead to additional musculoskeletal pain, postural instability, and an increased risk of falls or injuries [60]. Nevertheless, this evaluation should be supplemented by a comprehensive approach, encompassing gait analysis, assessment of joint mobility, and examination of footwear quality, as these factors are crucial in managing pain and enhancing functionality in FM patients. Improving foot function, which is often compromised in individuals with FM, could be essential in alleviating symptoms and enhancing the quality of life for those affected [36]. To address the identified problems, we propose the utilization of posturological analysis combined with manual therapies focused on muscle relaxation and postural correction. In our study, we have confirmed that muscle relaxation, together with increasing the foot bearing surface, contributes significantly to improved posture [37]. The findings indicate that manual therapy could enhance plantar pressure distribution in FM patients.

However, additional research is required to evaluate the long-term impacts and practicality of this intervention in clinical settings.

Manual therapy applied to the cervical and trunk muscles can influence plantar pressure distribution due to the relationship between myofascial chains and postural control [61]. In individuals with fibromyalgia (FM), postural alterations and muscle stiffness affect this distribution, leading to reduced functional performance [62]. From a biomechanical perspective, manual therapy aimed at relieving tension in the cervical and trunk muscles could optimize mobility in these structures [63]. This, in turn, could promote more efficient postural alignment and more balanced activation of the descending muscle chains that connect the trunk with the lower limbs. By reducing muscle tension through manual therapy, mobility, body alignment, and muscle balance can be improved, contributing to better posture. Some studies have already been published on the association between body posture and malocclusion in individuals without FM, which supports the idea of an integral connection between posture, foot placement, and body alignment [64,65].

A noteworthy observation was the impact of BMI on manual therapy outcomes. Participants with a ‘normal’ BMI exhibited more pronounced improvements in the variables analyzed compared to those categorized as overweight. This suggests that body weight might significantly influence the response to manual therapy, possibly due to the extra mechanical stress affecting plantar pressure distribution and overall biomechanics. These outcomes highlight the importance of considering BMI as a crucial factor when developing therapeutic interventions for FM patients, which is consistent with findings from other studies [66]. Moreover, recent research suggests that manual lymphatic drainage, when combined with medical treatment, could be an effective alternative for reducing pain intensity and improving the overall impact of FM [9]. This further supports the potential advantages of combining various therapeutic approaches to better manage FM symptoms and effects [67].

### Limitations and Recommendations

This study had some limitations that should be considered. First, the single-arm design and lack of a control group limit the ability to attribute causality directly to the intervention and make it difficult to distinguish between the specific effects of the intervention, placebo effect, and natural history of the condition. In addition, the sample size was relatively small, with only 24 participants, which could have affected the statistical power and generalizability of the results. We acknowledge that the sample size may limit the robustness of the statistical analyses and the ability to generalize our findings. Finally, the duration of the protocol might have been insufficient to observe changes in parameters peak pressure. Future research should include a randomized design with a control group and a larger sample size to confirm these findings. Implementing a randomized controlled trial (RCT) would enhance the robustness of the study design, allowing for a clearer interpretation of the intervention’s effectiveness and providing stronger external validity to the results of this study. In addition, it would be interesting to explore longer protocols that combine physiotherapy with manual therapy strategies, along with improvement of plantar pressure distributions, and evaluate their long-term impact on the functionality and quality of life of patients with FM.

## 5. Conclusions

This study indicates that manual therapy targeting the dorsal muscles in the lower back leads to changes in plantar surface distribution in patients with FM, particularly in terms of reducing the plantar contact surface. Although these findings suggest potential postural adaptations, no significant improvements in strength or peak pressure metrics were observed. The results highlight the potential influence of BMI on the extent of changes, with normal-weight participants showing more pronounced effects than those in the overweight category. Further research is needed to explore the long-term impact of these changes on physical functionality, pain management, and quality of life in this population, as well as to validate these preliminary findings through RCT with larger sample sizes.

## Figures and Tables

**Table 1:** Demographic data of the sample.

Outcome	All Participants (n = 24)	Overweight (n = 10)	Normality (n = 14)	*p*-Value
Age (years)	56.83 ± 6.89	58.77 ± 7.27	54.30 ± 5.76	0.18
Height (cm)	162.83 ± 4.76	162.00 ± 5.11	164.20 ± 4.18	0.28
Weight (Kg)	71.08 ± 9.19	77.29 ± 5.31	62.40 ± 5.62	0.001 *
Body mass index. (kg/m^2^)	26.87 ± 3.79	29.54 ± 2.28	23.12 ± 1.54	0.001 *
Shoe size	38.40 ± 1.05	38.21 ± 1.25	38.65 ± 0.67	0.33
Short Form-36 Health Survey	38.33 ± 14.87	38.00 ± 19.75	38.57 ± 10.99	0.52
P.Max Pre (gr/cm^2^)	1885.00 ± 352.66	1957.21 ± 329.36	1783.90 ± 376.39	0.24
P.Max Post (gr/cm^2^)	1811.00 ± 324.29	1918.71 ± 353.66	1660.20 ± 211.59	0.05 *
Surface Pre (cm^2^)	134.08 ± 32.28	142.14 ± 34.74	122.80 ± 26.03	0.15
Surface Post (cm^2^)	144.33 ± 33.24	157.93 ± 32.70	125.30 ± 24.28	0.01 *
RF Force Pre	50.00 ± 6.21	51.07 ± 5.51	48.50 ± 7.11	0.33
RF Force Post	49.75 ± 6.85	49.21 ± 7.26	51.07 ± 5.51	0.66
LF Force Pre	50.00 ± 6.21	48.93 ± 5.51	51.50 ± 7.11	0.33
LF Force Post	50.25 ± 6.85	50.79 ± 7.26	49.50 ± 6.54	0.66

Note. Student’s *t*-test. Statistically significant differences (*p* < 0.05) are marked with an asterisk (*).

**Table 2:** T-contrast for paired samples.

Measure 1	Measure 2	t	gl	*p*	D de Cohen	ET D de Cohen
Surface pre	Surface post	−3.01	23	0.006 *	−0.61	0.11
LF Surface pre	LF Surface post	−2.65	23	0.01 *	−0.54	0.14
LF Force pre	LF Force post	−0.20	23	0.85	−0.04	0.19
RF Surface pre	RF Surface post	−2.91	23	0.007 *	−0.59	0.10
RF Force pre	RF Force post	0.20	23	0.85	0.04	0.19
P.Max pre	P.Max post	1.25	23	0.22	0.25	0.18

Note. Student’s *t*-test/Degrees of freedom(gl). Statistically significant differences (*p* < 0.05) are marked with an asterisk (*).

**Table 3:** Paired sample BMI (normal/overweight) T-contrast for paired samples.

Pre	Post		t	gl	*p*	D de Cohen	ET D de Cohen
Surface	Surface	Normal	−3.45	13	0.004 *	−0.92	0.16
		Overweight	−0.60	9	0.57	−0.19	0.17
LF Surface	LF Surface	Normal	−3.45	13	0.004 *	−0.92	0.22
		Overweight	−0.08	9	0.94	−0.02	0.16
LF Force	LF Force	Normal	−1.42	13	0.18	−0.38	0.20
		Overweight	0.86	9	0.41	0.27	0.35
RF Force	RF Force	Normal	−2.98	13	0.01 *	−0.80	0.12
		Overweight	−0.98	9	0.35	−0.31	0.20
RF Force	RF Force	Normal	1.42	13	0.18	0.38	0.20
		Overweight	−0.86	9	0.41	−0.27	0.35
P.Max	P.Max	Normal	0.50	13	0.63	0.13	0.23
		Overweight	1.30	9	0.22	0.41	0.29

Note. Student’s *t*-test/Degrees of freedom(gl). Statistically significant differences (*p* < 0.05) are marked with an asterisk (*).

**Table 4:** Differences in surface and force (delta) between overweight and normal individuals.

	Overweight (n = 10)	Normal Range (n = 14)	*p*-Value
Delta Surface	15.79 ± 17.11	2.50 ± 13.25	0.05
Delta P.Max	−38.50 ± 289.72	−123.70 ± 300.06	0.49
Delta LF Surface	9.29 ± 10.06	0.20 ± 8.04	0.003
Delta RF Surface	6.50 ± 8.17	2.30 ± 7.42	0.21
Delta RF force	−1.86 ± 4.90	2.00 ± 7.35	0.14
Delta LF force	1.86 ± 4.90	−2.00 ± 7.35	0.14

Note. Contraste t de Student.

**Table 5:** BMI group delta.

	t	gl	*p*	D de Cohen	ET D de Cohen
Delta Surface	−2.05	22	0.05 *	−0.85	0.46
Delta P.Max	−0.70	22	0.49	−0.29	0.42
Delta P.Med	1.78	22	0.09	0.74	0.45
Delta PI Suface	−2.36	22	0.03 *	−0.98	0.47
Delta PD Surface	−1.29	22	0.21	−0.53	0.43
Delta PD Force	1.55	22	0.14	0.64	0.44

Note. Student’s *t*-test/Degrees of freedom(gl). Statistically significant differences (*p* < 0.05) are marked with an asterisk (*).

## Data Availability

The data presented in this study are available upon request to the corresponding author.

## Abbreviations

The following abbreviations are used in this manuscript:

Abbreviation	Definition
FM	Fibromyalgia
MT	Manual Therapy
BMI	Body Mass Index
PCS	Physical Component Summary
MCS	Mental Component Summary
RFS	Right Foot Surface
LFS	Left Foot Surface
P.Max	Max Pressure
P.Med	Media Pressure
PD	Right Foot
PI	Left Foot
NCT	National Clinical Trial
SPSS	Statistical Package for the Social Sciences
JASP	Jeffreys’s Amazing Statistics Program
ET	Effect Size
ACR	American College of Rheumatology
CGCOP	General Council of Official Colleges of Podiatry
